# The contribution of mitochondria-associated endoplasmic reticulum membranes (MAMs) dysfunction in Alzheimer’s disease and the potential countermeasure

**DOI:** 10.3389/fnins.2023.1158204

**Published:** 2023-03-07

**Authors:** Zehui Li, Yu Cao, Hui Pei, Lina Ma, Yang Yang, Hao Li

**Affiliations:** ^1^Department of Geriatrics, Xiyuan Hospital, China Academy of Chinese Medical Sciences, Beijing, China; ^2^Wangjing Hospital, China Academy of Chinese Medical Sciences, Beijing, China

**Keywords:** Alzheimer’s disease, mitochondria, endoplasmic reticulum, calcium homeostasis, lipid metabolism

## Abstract

Alzheimer’s disease (AD) is the most common neurodegenerative disease. There are many studies targeting extracellular deposits of amyloid β-peptide (Aβ) and intracellular neurofibrillary tangles (NFTs), however, there are no effective treatments to halt the progression. Mitochondria-associated endoplasmic reticulum membranes (MAMs) have long been found to be associated with various pathogenesis hypotheses of AD, such as Aβ deposition, mitochondrial dysfunction, and calcium homeostasis. However, there is a lack of literature summarizing recent advances in the mechanism and treatment studies. Accordingly, this article reviews the latest research involving the roles of MAM structure and tethering proteins in the pathogenesis of AD and summarizes potential strategies targeting MAMs to dissect treatment perspectives for AD.

## Introduction

According to Alzheimer’s disease (AD) International, 55 million people worldwide suffer from dementia. The most common type of dementia is AD. The main histopathological characteristics of AD are the accumulation of extracellular deposits of amyloid β-peptide (Aβ) and intracellular neurofibrillary tangles (NFTs). However, there are other biochemical and morphological characteristics present earlier in the course of AD, such as alterations in phospholipid metabolism, the elevation of circulating cholesterol levels, aberrant calcium regulation, reduction of brain glucose levels, and mitochondrial dysfunction. Plaques and tangles have received a lot of attention because they are observable physical entities, however, the other features may be the upstream factors and should not be overlooked (Area-Gomez and Schon, 2017).

Mitochondria-associated endoplasmic reticulum membranes (MAMs) are a special subdomain of the endoplasmic reticulum (ER) that physically and biologically connects mitochondria to ER. ER-mitochondrial communication and MAM functions are increased expressively in AD (Leal et al., 2020). MAMs are generated by the ER side-by-side to the mitochondrial outer membrane linked by a series of tethering proteins (Degechisa et al., 2022). They are not the collection of membranes but the employment of the proteinaceous tethers (Rowland and Voeltz, 2012), which play a key role in many important metabolic processes including the transfer of calcium, mitochondrial dynamics, lipid synthesis, autophagy, apoptosis, and inflammation. MAM dysfunction is also central to the pathogenic mechanisms of AD, especially in the Aβ generation and deposition, mitochondrial dysfunction, imbalanced calcium homeostasis, abnormal lipids metabolism, and abnormal autophagy. This review highlights abnormal MAM structures and tethering proteins in the pathogenesis of AD from these aspects. In addition, we provide a summary of compounds and drugs that target MAM tethering proteins in AD models.

## The role of MAMs in Aβ generation of AD

The Aβ peptide is the main component of the AD hallmark amyloid plaques, which is produced by the proteolysis of amyloid beta-precursor protein (APP) by two enzymes: β-site APP cleaving enzyme 1 (BACE1) and the γ-secretase complex. Many studies have shown that MAMs are the main site of Aβ generation. Schreiner et al. (2015) reported that Aβ may be generated directly in MAMs. APP, BACE1, and γ-secretase have all been found in MAMs (Leal et al., 2020). BACE1 cleaves APP to produce sAPPβ and C99. C99 is delivered to MAMs and cleaved to produce Aβ and APP intracellular domain (AICD) by γ-secretase (Montesinos et al., 2020). In addition, it has been reported that the main pathway for Aβ entering mitochondria is MAMs (Del Prete et al., 2017). Knockdown of mitofusin-2 (Mfn2), which is involved in MAM tethering, leads to decreased contact between mitochondria and ER, resulting in lower γ-secretase activity and decreased concentrations of intracellular and extracellular Aβ_40_ and Aβ_42_ (Leal et al., 2016). This proves that the increase of MAMs may promote mitochondrial Aβ deposition. Axonal generation of Aβ also plays a key role in AD pathology. In AD models, swollen axons contain high levels of BACE1 (Gowrishankar et al., 2017). An average of 37 ± 4% of the total Aβ_40_ secreted from each mouse hippocampal neuron is secreted by axons (Niederst et al., 2015). Bhattacharyya et al. showed that down-regulation of MAM assembly by silencing MAM-resident sigma-1 receptor expression resulted in reduced palmitoylated APP cleavage by BACE1, thereby decreasing Aβ generation in neuronal processes and axons (Bhattacharyya et al., 2021).

## The role of MAMs in mitochondria dysfunction of AD

The neuronal activity must depend on the energy produced by mitochondria. Mitochondrial death can be observed before the histopathological features of AD appear. MAMs wrap around the location where mitochondria undergo fissuring by recruiting MAM protein inverted formin 2 (INF2) (Steffen and Koehler, 2018). The mitochondrial outer membrane protein FUNDC1 is a MAM protein that recruits dynamin 1 Like (DNM1L)/dynamin-related protein 1 (DRP1) to drive mitochondrial fission (Wu et al., 2016). In an AD mouse model, alterations in MAMs precede changes in mitochondrial dynamics accompanied by aberrations in mitochondrial membrane potential (MMP) and ATP production (Leal et al., 2020). Moreover, the MAMs control mitochondrial Ca^2+^ intake. Reduced Ca^2+^ intake affects mitochondrial metabolism, leading to mitochondrial dysfunction. Fernandes et al. (2021) found a decrease in close ER-mitochondria contacts, a reduction of Ca^2+^ transfer from ER to mitochondria, and impaired mitochondrial function in APPswe cells. γ-secretase activating protein (GSAP) fluorescence staining showed high colocalization with a MAM marker Facl4. Knockdown of GSAP significantly increased mitochondrial basal respiration and total ATP levels, which suggests that GSAP impairs mitochondria function (Xu et al., 2021). In an AD cell model, increased concentration of unprocessed C99 in the MAM region increased sphingolipid turnover and altered the lipid composition of mitochondrial membranes, which can interfere with the normal activity of the respiratory supercomplexes and thus may contribute to the bioenergetic defects in AD (Pera et al., 2017). Atlastin 2 (ATL2) is a protein associated with ER-mitochondria contacts whose expression was significantly increased both in 3 × Tg mice and AD patients. While, knockout of ATL2 rescued the elevated ER-mitochondria contacts back to normal levels, reduced the abnormally elevated mitochondrial superoxide, and significantly increased the MMP (Han et al., 2021).

## The role of MAMs in calcium homeostasis of AD

Calcium signaling in neurons is essential for neurotransmission and the maintenance of synaptic plasticity (Skobeleva et al., 2022). Dysregulation of calcium homeostasis disrupts neuronal and synaptic function in AD (McDaid et al., 2020). Ca^2+^ homeostasis depends on the Sarcoplasmic/endoplasmic reticulum Ca^2+^ ATPase (SERCA) pumps to regulate Ca^2+^ uptake from the cytoplasm to the ER and activated inositol 1,4,5-triphosphate receptors (IP3Rs) and ryanodine receptors (RyRs) to enable Ca^2+^ efflux from the ER (Kawamoto et al., 2012). The IP3R–glucose-regulated protein 75 (Grp75)–voltage-dependent anion channel (VDAC) Ca^2+^ channeling complex was concentrated in MAMs. Up-regulation of this complex results in increased calcium content. The accumulation of mitochondrial calcium could be prohibited by the IP3R inhibitor (Chen et al., 2021b). The RyR is a Ca^2+^-release channel protein located in the MAMs and sarcoplasmic reticulum. The RyR3 plays a dual character in AD pathology. Deletion of RyR3 in young APP/PS1 mice increases the excitability of hippocampal neuronal networks and accelerates AD progress. However, in aged APP/PS1 mice, the deletion of RyR3 restored network excitability (Liu et al., 2014). In the 5xFAD mouse model of AD, restricting RyR2 open time blocked the excessive activity of CA1 neurons (Yao et al., 2020). A small-molecule SERCA activator can increase ER Ca^2+^ and has shown efficacy in APP/PS1 mice, supporting SERCA activation as a therapeutic strategy for AD (Krajnak and Dahl, 2018).

## The role of MAMs in lipid metabolism of AD

Lipid metabolism, especially cholesterol metabolism, has been implicated in the synaptic dysfunction of AD (Petrov et al., 2017). Ceramide and cholesterol have been found to increase in the brains of AD patients, normal aging mice, and neurons exposed to Aβ_1–42_ (Cutler et al., 2004). MAMs are transient lipid rafts that are closely related to cholesterol and phospholipid metabolism (Pera et al., 2020). It has been proven that phospholipid transport between the ER and mitochondria is dependent on membrane integrity including MAMs rather than energy or MMP (Kojima et al., 2016). MAMs are where the enzymatic activities such as acetyl-CoA acetyltransferase 1 (ACAT1) that regulate cholesterol levels reside. ACAT1 gene ablation led to the amelioration of amyloid pathology and cognitive deficits in 3 × Tg AD mice (Bryleva et al., 2010). The concentration of unprocessed C99 at the MAMs is increased in models of AD and cells from AD patients, subsequently associated with the increase in ceramide levels (Pera et al., 2017). GSAP enriched in MAMs regulates lipid homeostasis through the processing of APP (Xu et al., 2021). In a cortical impact mice model of AD, the injured cortex and hippocampus exhibited significant increases in MAM activity, phospholipid synthesis, sphingomyelinase (SMases) activity, and cholesterol turnover (Agrawal et al., 2022). When cholesterol concentrations exceed a certain threshold, SMases are activated and hydrolyze sphingomyelin (SM) to produce ceramides. The cholesterol affinity of APP is involved in limiting APP distribution, conversely, APP senses and balances the membrane cholesterol (DelBove et al., 2019). An increase in MAM-localized C99 triggers the upregulation of SMase activity. The increased cholesterol mobilization observed in AD cells may be an outcome of persistent SMase activity caused by increased MAM-C99, which disrupts cellular lipid homeostasis and causes the alterations in membrane lipid composition commonly happen in AD (Montesinos et al., 2020).

## The role of MAMs in abnormal autophagy in AD

**FIGURE 1:**
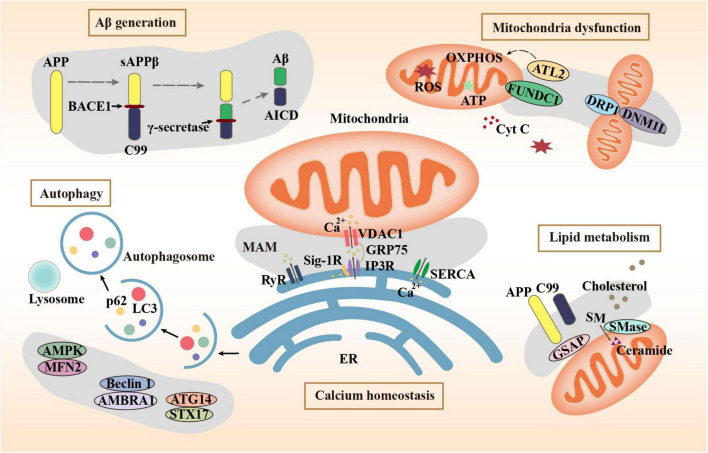
Mechanisms related to MAMs in AD.

Autophagy is a degradation mechanism of cells to remove damaged and senescent organelles and maintain cellular homeostasis. Autophagy plays a vital role in the generation and clearance of Aβ. Abnormal autophagy is involved in the development of AD (Yuan et al., 2022). Activation of autophagy alleviates pathological characters and cognitive deficits in APP/PS1 mice (Yang et al., 2023). The MAMs mark the starting point of autophagosome formation. Mitochondrial fusion protein Mfn2 is a MAM protein responsible for binding the ER and mitochondria. The energy sensor AMP-activated protein kinase (AMPK) interacts with Mfn2 and phosphorylates Mfn2 and induces autophagy (Hu et al., 2021). Autophagy initiation proteins such as autophagy and beclin 1 regulator 1 (AMBRA1) and Beclin 1 are recruited to the MAMs to regulate autophagy, demonstrating that MAM raft-like microdomains play a crucial part in the autophagosome formation (Garofalo et al., 2016). The sigma-1 receptor (Sig-1R) is a receptor with molecular chaperone activity clustered on MAMs. Autophagosome clearance is impaired in Sigma-1 KO cells, possibly due to impaired autophagosome-lysosomal organelle fusion mediated by the complex formed by Sig-1R with STX17 and ATG14. STX17 and ATG14 both appear at the ER-mitochondria contact site (Yang et al., 2019). The combination of vesicle-associated membrane protein-associated protein B (VAPB) and protein tyrosine phosphatase interacting protein 51 (PTPIP51) is identified as one of the MAM tethers. Manipulation of their expression to increase and decrease ER-mitochondrial junctions had significant effects on autophagy (Gomez-Suaga et al., 2017). The decreased expression of MAM tethering protein complexes including VAPB-PTPP51, BAP31-FIS1, and Mfn2-Mfn1 leads to abnormal autophagy, which leads to the decline of cognitive ability (Liu et al., 2022). The role of MAM tethering proteins in several major pathogeneses of AD is summarized in Figure 1 schematically.

## Strategies targeting MAMs for treating AD

The functions of MAMs are mainly exerted by its numerous tethering proteins. The abnormalities of these proteins eventually lead to the pathological processes of AD through multiple mechanisms. The expression of MAM tethering proteins and the tight connection between mitochondria and ER can be one of the hallmarks of AD progression. The regulation of these proteins to improve the corresponding phenotypes can be used as a strategy and target for AD treatment.

Knockdown of MAM protein GSAP reduced Aβ generation and plaque formation in an AD mouse model (He et al., 2010). Imatinib and imatinib methanesulfonate could prevent Aβ formation by inhibiting GSAP (He et al., 2010; Weintraub et al., 2013; Chu et al., 2014). Lithium treatment reduced abnormal IP3R-dependent ER Ca^2+^ signaling and enhanced synaptic plasticity in 3 × Tg AD mice (Wiseman et al., 2023). Xestospongin C can ameliorate the Ca^2+^ overload of primary hippocampal neurons induced by Aβ_1–42_ and improve the cognitive ability of APP/PS1 mice (Wang et al., 2019). Mecobalamin also reduces ER-mitochondrial calcium flux through IP3R and prevents mitochondrial dysfunction (Wang and Xu, 2019). RyR2 instability plays a vital role in the reduction of ER Ca^2+^ content, which alters synaptic transmission and plasticity mechanisms. While Dantrolene stabilizes RyR2 thereby reversing most AD-related phenotypes in AppNL-G-F mice (Nakamura et al., 2021). Compound 12a inhibited Ca^2+^ release and significantly accelerated the cognitive behavior of FAD mice in the Morris water maze test. Moreover, docking simulations testified that 12a could bind to the active site of RyR1 (Dai et al., 2021). A Sig-1R agonist (+) SKF-10,047 could significantly increase mitochondrial movement in cortical neurons of 3 × Tg mice, which might be because it leads to the removal of Sig-1R from MAMs to mitochondria. (+) SKF-10,047 also leads to an increase in the number of mitochondria (Cavendish et al., 2019). Two other Sig-1R agonists ANAVEX2-73 and PRE-084 could prevent mitochondrial respiratory dysfunction in Aβ_25–35_-injected mice (Lahmy et al., 2014). IRE1α inhibitor 4μ8c can reduce ER-mitochondrial association and restore the normal function of MAMs by inhibiting the expression and interaction of IP3R, Grp75, and VDAC1, thereby restoring ATP content and MMP (Chu et al., 2021). Artesunate could reverse cognitive impairment in APP/PS1 mice by regulating DRP1 to maintain mitochondrial dynamics (Qin et al., 2022). By increasing the expression of the MAM protein Mfn2, Biochanin A could reverse the imbalance of mitochondrial dynamics and abnormal mitophagy in APP/PS1 mice (Hou et al., 2022). Curcumin restores basal mitochondrial respiration and ATP production in thapsigargin-injured SH-SY5Y cells by targeting Mfn2 (Zhou et al., 2022). Besides, Myricetin, Selenomethionine, Leptin, *Trans*-ferulic acid, Ligustilide, and Albiflorin were also able to regulate Mfn2 and ameliorate mitochondrial dysfunction (Xu et al., 2018, 2019; Zafeer et al., 2019; Cheng et al., 2020; Zhu et al., 2020; Chen et al., 2021a; Yao et al., 2022). Cholesteryl ester produced in MAMs is involved in the pathogenesis of AD. Progesterone reduced the expression of ACAT1 through the ERK1/2 pathway, shortened the abnormally prolonged MAM length, inhibited cholesteryl ester accumulation in the cortex, and improved the cognitive function of APP/PS1 mice (Shi et al., 2021). We summarize potential compounds and drugs that exert anti-AD effects by regulating MAM tethering proteins in Table 1.

**TABLE 1:** Compounds and drugs targeting MAM tethering proteins for treating AD.

Compound/drug	Target	Mechanism	Model	References
Imatinib	GSAP	Aβ generation	3 × Tg mice N2a-APP695 cells	He et al., 2010; Chu et al., 2014
Imatinib methanesulfonate	GSAP	Aβ generation	LPS-induced inflammation	Weintraub et al., 2013
Lithium	IP3R	Ca^2+^ signaling abnormalities	3 × Tg mice	Wiseman et al., 2023
Xestospongin C	IP3R	Ca^2+^ homeostasis	APP/PS1 mice, Aβ_1–42_-treated primary hippocampal neurons	Wang et al., 2019
Methyl vitamin B12	IP3R	Ca^2+^ homeostasis, mitochondrial dysfunction	Aβ-treated PC12 cells	Wang and Xu, 2019
Dantrolene	RyR2	Ca^2+^ homeostasis	App^NL–G–F^ mice	Nakamura et al., 2021
12a	RyR1	Ca^2+^ homeostasis	FAD mice	Dai et al., 2021
(+) SKF-10,047	Sig-1R	Mitochondrial movement and number	Primary hippocampal neurons from 3 × Tg mice	Cavendish et al., 2019
ANAVEX2-73, PRE-084	Sig-1R	Mitochondrial respiratory dysfunction	Aβ_25–35_-injected mice	Lahmy et al., 2014
4μ8c	IP3R, Grp75, and VDAC1	Mitochondrial dysfunction	Aβ-treated SH-SY5Y cells	Chu et al., 2021
Artesunate	DRP1	Mitochondrial dynamics	APP/PS1 mice	Qin et al., 2022
Biochanin A	Mfn2	Mitochondrial dynamics and mitophagy	APP/PS1 mice	Hou et al., 2022
Curcumin	Mfn2	Mitochondrial dysfunction	Thapsigargin-treated SH-SY5Y cells	Zhou et al., 2022
Myricetin	Mfn2	Mitochondrial dysfunction	N2a-APP695-Swedish cells	Yao et al., 2022
Selenomethionine	Mfn2	Mitochondrial dysfunction	N2a-APP695-Swedish cells, 3 × Tg mice	Chen et al., 2021a
Leptin	Mfn2	Mitochondrial dysfunction	Aβ1-42-treated SH-SY5Y cells	Cheng et al., 2020
*Trans*-ferulic acid	Mfn2	Mitochondrial dysfunction	Streptozocin injection	Zafeer et al., 2019
Ligustilide	Mfn1, Mfn2	Mitochondrial dysfunction	SAMP8 mice APP/PS1 mice	Xu et al., 2018; Zhu et al., 2020
Albiflorin	Mfn1, Mfn2	Mitochondrial dysfunction	APP/PS1 mice	Xu et al., 2019
Progesterone	ACAT1	Cholesterol metabolism	APP/PS1 mice	Shi et al., 2021

## Discussion

Drug development for AD has continuously been challenged. The most popular AD hypotheses are amyloid cascade, hyperphosphorylation of tau, and mitochondrial cascade. However, numerous studies targeting these hypotheses have not been able to fully elucidate the mechanism of AD or retard its progression. The interaction between different organelles, especially mitochondria and ER in cells has gradually emerged in the study of various diseases (Theurey and Rieusset, 2017; Luan et al., 2021). Cellular dysfunction in the early stage of AD, including Ca^2+^ homeostasis, mitochondrial dysfunction, oxidative stress, and abnormal autophagy, are all associated with MAM function (Tapella et al., 2022).

As for the amyloid cascade, MAMs are the sites where C99 is cleaved to Aβ. Inhibition of MAMs protein expression and function may reduce Aβ production and extracellular deposition. MAMs recruit proteins such as INF2 and DRP1 to participate in mitochondrial fission, thereby affecting MMP and ATP production. Upregulation of some MAM-resident proteins causes enhanced mitochondria-ER contacts, along with mitochondrial damage such as increased mitochondrial superoxide. The MAM has both protein pumps that allow Ca^2+^ to flow from the cytoplasm to the ER and Ca^2+^ to flow from the ER, thus regulating Ca^2+^ homeostasis. Ca^2+^ flux also plays a dual role in AD progression. For instance, excessive Ca^2+^ influx leads to increased ROS production and cell death due to caspase activation; however, attenuated Ca^2+^ signaling may also be detrimental to ATP production (Filadi and Greotti, 2021; Arnst et al., 2022). Modulation of MAM-resident enzymes such as ACAT1 and MAM-C99 content affects cholesterol levels and lipid homeostasis in AD, along with amyloid synthesis and synaptic transmission. MAMs mark the starting point of autophagy. Decreased expressions of MAM tethering proteins, such as Sig-1R, VAPB-PTPP51, and Mfn2-Mfn1, lead to abnormal autophagy. All these indicate that MAM is an important target that should not be ignored in the study of AD pathogenesis.

Although MAMs have been shown to be closely related to the progression of AD, the molecular pathways are still not fully understood. At present, the research targets of AD drugs targeting MAM proteins are still limited. The mechanisms of interest are mainly in Aβ production, mitochondrial function, calcium homeostasis, and lipid metabolism. Currently, there are few studies on the interaction between MAMs and tau phosphorylation. Besides, although tethering proteins are the main manifestation of MAM function, the observation of MAM structure should not be ignored despite the technical difficulties in the research of AD treatment strategies. We look forward to new studies to further explore the role of the structure and functions of MAMs in the pathogenesis and treatment of AD.

## Author contributions

YY and HL conceived the idea. ZL wrote the manuscript. YC, HP, and LM revised the manuscript. All authors contributed to the work and approved the submitted version.
